# Karyotyping and prenatal diagnosis of 47,XX,+ 8[67]/46,XX [13] Mosaicism: case report and literature review

**DOI:** 10.1186/s12920-019-0639-8

**Published:** 2019-12-21

**Authors:** Shaohua Sun, Fang Zhan, Jiusheng Jiang, Xuerui Zhang, Lei Yan, Weiyi Cai, Hailiang Liu, Donghua Cao

**Affiliations:** 1 Genetic Disease Laboratory, Dalian Maternal and Child Health Care Hospital, Dalian, 116033 China; 2 CapitalBio Technology Inc, Beijing, 101111 China

**Keywords:** Trisomy 8 mosaicism, Karyotyping and prenatal diagnosis, Next generation sequencing (NGS)

## Abstract

**Background:**

Trisomy 8 mosaicism has a wide phenotypic variability, ranging from mild dysmorphic features to severe malformations. This report concluded a female pregnant woman with trisomy 8 mosaicism, and carefully cytogenetic diagnoses were performed to give her prenatal diagnostic information. This report also provides more knowledge about trisomy 8 mosaicism and the prenatal diagnostic for clinicians.

**Case presentation:**

In this present study, we reported one case of pregnancy woman with trisomy 8 mosaicism. Noninvasive prenatal testing prompted an abnormal Z-score, but further three dimension color ultrasound result suggested a single live fetus with no abnormality. The phenotypic of the pregnant woman was normal. Based on our results, there were no abnormal initial myeloid cells (< 10^− 4^), which suggested that the patient had no blood diseases. The peripheral blood karyotype of the patient was 47,XX,+ 8[67]/46,XX [13], and karyotype of amniotic fluid was 46, XX. The next generation sequencing (NGS) result suggested that the proportions of trisomy 8 in different tissues were obviously different; and 0% in amniotic fluid. Last, the chromosomes of the patient and her baby were confirmed using chromosome microarray analysis (CMA), and the results were arr[GRCh37](8) × 3,11p15.5p13(230750–33,455,733) × 2 hmz and normal.

**Conclusions:**

This pregnancy woman was trisomy 8 mosaicism, but the phenotypic was normal, and also the fetus was normal. Carefully cytogenetic diagnoses should be performed for prenatal diagnose.

## Background

Trisomy syndrome is the most common type of chromosome (Chr) abnormality in humans. Multiple studies have shown that 90% of trisomy syndrome cases involve Chr2, 13, 15, 16, 21, 18, 22 and X and that more than 95% happens aborted before birth; furthermore, multiple cytogenetic reports have indicated that trisomies related to chromosomes 2, 16 and 22 almost always affect placental tissue rather than the fetuses (Confined placental mosaicism) [1]. Among them, constitutional trisomy 8 mosaicism syndrome (T8MS), also known as Warkany syndrome, is a rare viable condition reported in 1/25,000 to 50,000 of live births and is more prevalent in males than in females (5:1) [2]. Trisomy 8 is a rare condition in humans, constituting 0.7% of spontaneous abortions, and is estimated to occur in approximately 0.1% of recognized pregnancies. Chr. 8 is completely or partially duplicated in various hematologic diseases, especially myeloid diseases, and is relatively common in M4, M5, and CMML [3]. In addition to myeloid blood diseases, the clinical characteristics for trisomy 8 include retardation, language barriers, webbed neck, movement disorders, horseshoe varus foot, skull dysplasia, oral ulcers, repeated cases of colon ulcers, arthritis, strabismus, auricle small, full palm, and developmental abnormalities of the heart, bone, kidney and the central nervous system [4]. The characteristic phenotypic features of mosaic trisomy 8 are widely variable, and deep furrows on the soles of the feet are highly characteristic [5–7].

Trisomy 8 mosaicism is an uncommon disorder, and the prenatal detection of trisomy 8 mosaicism can lead to problems in genetic counselling. So, in the present study, we report one rare case of a pregnancy woman who was diagnosed trisomy 8 mosaicism, and we used a variety of methods to perform a careful prenatal cytogenetic diagnosis for her and obtain more knowledge, which could reference for T8 and prenatal diagnoses for clinicians.

### Case presentation

This study was approved by Ethics Committee of Dalian Maternal and Child Health Hospital, and the informed consent signed by the patient.

The patient was female, was 37 years old, was from Liaoning province in China, and had been examined at our hospital at 13 weeks of pregnancy. The color ultrasound results showed that the nuchal translucency (NT) value was 2.4 mm, and NIPT using an Ion Proton sequencer (CapitalBio Technology Inc., Beijing) at 16 gestational weeks was performed. However, the Z-score(102.835) for Chr8 was outside the normal range and suggestive of trisomy 8. Because most cases of complete trisomy 8 are spontaneously aborted in the early stages of pregnancy, we highly suspected that trisomy 8 was from the mother and that trisomy 8 was mosaic. Further examination by three-dimensional color ultrasound suggested a single live fetus with no abnormalities. The patient denied smoking, drinking or radiation, and was without chemical exposure. We evaluated the brain development and reproductive system of the fetus by ultrasound, there was no obvious abnormal clinical manifestations, and the fetus had developed normally. The patient had previously been pregnant three times, resulting in one cesarean section, one spontaneous abortion, and one birth to a girl who is currently 8 years old and in good health.

The peripheral blood from the patient was extracted for hemocyte classification analysis and smear microscopy detection. We found no abnormal juvenile cells in the peripheral blood by smear microscopy. Through the detection of leukocyte immune typing, we found that CD117^+^/CD34^+^ cells accounted for 0.30% of nuclear cells, that these cells were all normal myeloid primitive cells, and that granulocytes accounted for 41.46% of nuclear cells. Immature monocytes occupied 0.02% of nuclear cells (0.53% of monocytes). Bone marrow stem cells, B cells, T cells, and NK cells were normal, and no abnormal myeloid primitive cells (< 10^− 4^) were observed in this clinical report. In addition, the development model for cells was normal (Tables 1 and 2).

**Table 1:** The patient blood routine results

ITEM	Result	Reference value
White blood cell count	5.68	3.5–9.5*10^9/L
Hemoglobin number	118	115-150 g/L
Red blood cell count	3.29	3.8–5.1*10^12/L
platelet	155	125–350^9*10/L
Percentage of neutrophils	68.4	40–75%
Absolute neutrophils	3.89	1.8–6.3*10^9/L
Percentage of lymphocytes	21.7	20–50%
Lymphocyte absolute value	1.23	1.1–3.2*10^9/L
Mononuclear cell percentage	9.3	3–10%
Absolute value of mononuclear cells	0.53	0.1–0.6*10^9/L
Percentage of eosinophils	0.4	0.4–8%
Absolute value of eosinophils	0.02	0.02–0.52*10^9/L
Percentage of basophils	0.2	0–1%
Basophil absolute value	0.01	0–0.06*10^9/L
Platelet volume distribution width	8.7	10-20 fl
Mean platelet volume	8.9	7.6–13.2 fl
thromboembolism	0.14	0.1–0.5%
hematocrit	33.6	37–52%
Mean erythrocyte volume	105	82-100 fl
Mean red blood cell hemoglobin content	36.9	27-34 pg
Average hemoglobin concentration	351	316-354 g/L
Erythrocyte volume distribution width-sd	57.1	39-46 fl
Erythrocyte volume distribution width -SV	14.9	10.9–15.4%
Macroplatelet ratio	16.7	13–43%

**Table 2:** Results of coagulation function in patients

ITEM	Result	Reference value
Prothrombin time	10.5	9-13S
International standardized ratio (PT)	0.91	0.8–1.2
Prothrombin temporal activity	115	70–150%
Activation time of partial thrombin	23.5	20-40S
Thrombin time	15.5	14-21S
fibrinogen	3.83	2-4 g/L

**Fig. 1:**
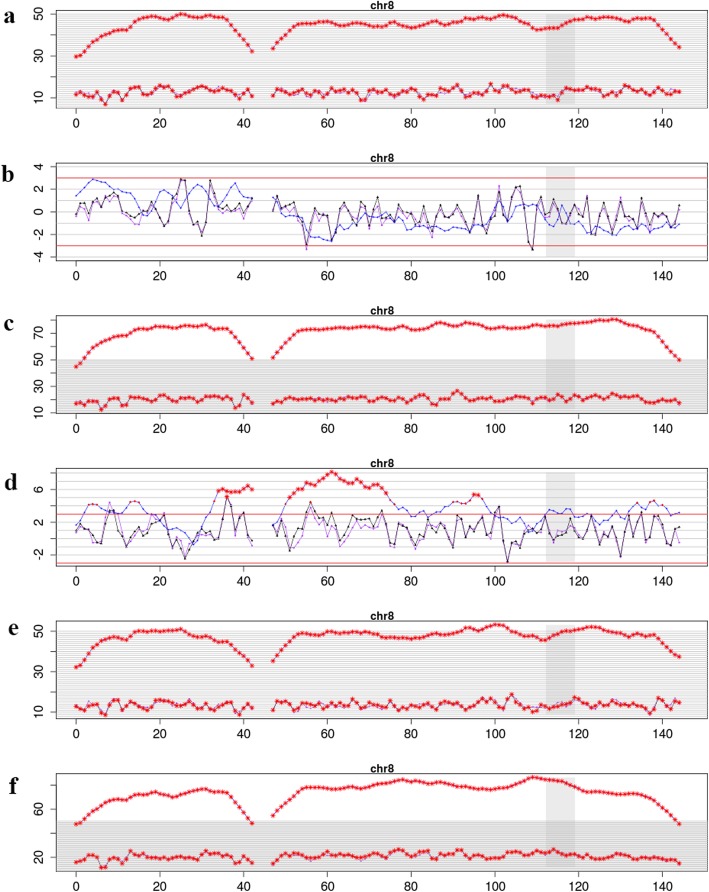
NIPT and NGS results for the fetal and patient. **a** analysis of fetal karyotype by NIPT, the Z score was 102.835; **b** analysis of fetal amniotic fluid karyotype by NGS, the Z score was − 0.051 and the predicted value of T8M was 0%; **c** about the patient blood karyotype by NGS, the Z score was 158.487 and the predicted value of T8M was 100%; **d** Chromosome analysis of buccal tissue samples from the patient, the Z score was 8.246 and the predicted value of T8M was 5%; **e** Chromosome analysis of saliva samples from the patient, the Z score was 108.81 and the predicted value of T8M was 60%; **f** Chromosome analysis of cervical exfoliated cells from the patient, the Z score was 161.646 and the predicted value of T8M was 100%

**Fig. 2:**
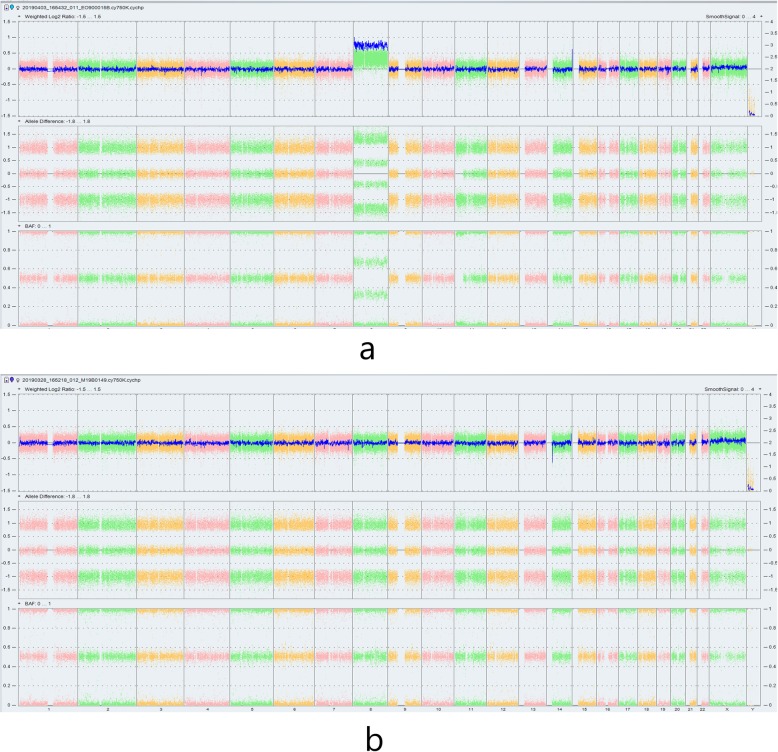
CMA assay results for the female patient and her baby. **a** CMA result of the pregnant woman, arr[GRCh37](8) × 3,11p15.5p13(230750–33,455,733) × 2 hmz. **b** CMA result of her baby, normal

Then, the peripheral blood from the patient, her husband and her daughter and amniotic fluid from the patient were collected to undergo chromosome G band analysis. From these results, we knew that the peripheral blood karyotype of the patient was 47,XX,+ 8[67]/46,XX, while the amniotic fluid karyotype was 46,XX; the karyotypes for her husband and daughter were 46,XY, and 46,XX, respectively. In addition, trisomy 8 mosaicism was confirmed using Ion Proton sequencer (CapitalBio Technology Inc., Beijing) at 400 flows according to the manufacturer’s instructions. The amniotic fluid, pharynx, cheek, saliva and cervical abscission cells samples from the patient were undergone next generation sequenced. The retained reads were aligned to the human genomic reference sequences (hg19) using the BWA. The fetal DNA concentration was calculated as a quality control, as described in Yin’s paper [8]. Combined GC-correction and Z-score testing methods were used to identify fetus autosomal aneuploidy for trisomy, as described in Liao’s paper [9]. A Z score range from − 3 to 3 was considered to indicate a low risk for a trisomy chromosome, and if Z score was > 3, the sample was in the high-risk zone. The result was considered as a trisomy when the Z-score was far higher than the normal value and when the average score of the black curve was in + 20. When the Z-score was far below the normal value and when the average score of the black value curve was − 20, a monomer was considered. Chromosomal chimerism was considered 1) when the Z-score was greater than or less than plus or minus 3 but far less than the Z-score of complete trisomer or greater than the Z-score of the complete and 2) when the mean score of the black curve was greater than or less than 0 but far less than the mean value of the complete trisomer or greater than the mean value of the complete monomer. Chimeric proportion (%) = Z-score range /0.2/100 (female) or Chimeric proportion (%) = Z-score range /0.2/100/concentration of the sample (male). Moreover, NGS result suggested that the proportions of trisomy 8 in different tissues were obviously different (Fig. 1). The proportions for trisomy 8 in cheek, saliva and cervical abscission cells samples were 5, 60, and 100%, respectively. In addition, the amniotic fluid was 0%, which suggested that the fetus was normal (Fig. 1). Last, the chromosomes of the patient and her baby were further examined using chromosome microarray analysis (CMA) with the higher resolution CytoScan® HD platform (Affymetrix, Santa Clara, CA) (Fig. 2). The CMA analysis showed that the chr.8 detection result in the plasma of the patient was arr[GRCh37](8) × 3,11p15.5p13(230750–33,455,733) × 2 hmz, and the result of her baby was normal.

## Discussion and conclusions

Chromosome 8 is an autosomal chromosome of medium length in humans that includes 793 gene and 301 pseudogene, and this chromosome contains 701 nonrepeating proteins according to Swiss-Port [10]. Eight percent of these genes on chromosome 8 are related to neurodevelopment and function, and 6% are related to cancer. The mutation rate of chromosome 8 is high, and the instability of chromosome 8 is mainly associated with the multiple diseases that are caused by gene mutations, such as the occurrence, mutation, and metastasis of tumor. To date, studies on chromosome 8 have been conducted in cancers related to diseases of the digestive system and hematological system. Trisomy 8 has been identified in malignant diseases of the blood, especially myelodysplastic syndrome (MDS), acute myelocytic leukemia (AML), and chronic myelogenous leukemia (CML). Trisomy 8 plays an important role in the development of myeloid disease. Most of trisomy 8 patients display mosaicism. In addition to malignant diseases of the blood, the clinical symptoms of trisomy 8 mosaicism include abnormal physical growth and mental retardation. In the present study, the NIPT result indicated high suspicion of trisomy 8 in the pregnant woman, and further testing of different tissues confirmed that the proportions of trisomy 8 were different. However, the leukocyte immune typing test indicated that there was no hematological disease and no clinical manifestation in the patient.

Chromosome trisomy syndrome formation occurs when chromosomes do not separate in the process of meiosis in which gametes are formed, and this syndrome correlates with multiple variables, including the age of the pregnant woman, virus infection, and other adverse physical and chemical factors [11]. The proportions of cells affected by the chromosome trisomy in different tissues are closely related to the incidence and severity of the abnormality and degree of mental retardation. Thus, the characteristic phenotypic features in mosaic trisomy 8 show wide variability. Prediction of the phenotype is difficult, since clinical severity is not related to the level of mosaicism [11, 12]. Malformations, including corpus callosum agenesis and renal abnormalities, have been described by many studies in subjects with trisomy 8 mosaicism [7], Most cases of complete trisomy 8 are generally spontaneously aborted in the early stages of pregnancy. Universal trisomy 8 is lethal and accounts for 0.7–0.8% of spontaneous abortions [13]. However, individuals with cognitive development within the normal range have also been reported in the literature, as described in Kurtyka’s paper [14] and in our case.

Trisomy 8 mosaicism occurs due to non-disjunction of chromosome 8 during mitosis in the zygote phase of fetal development. This condition is clinically heterogeneous and is associated with wide range of clinical abnormalities [15], and most trisomy 8 patients display mosaicism types. It was suggested by Mark [16] that the basis of this phenotypic variability may be the presence of different proportions of trisomy 8 cells in different tissues of the body. Depending on the tissue and proportion of the tissue that contains the trisomic clone, different specific phenotypes can be observed. As previously reported, one case trisomy 8 patient has no obvious clinical symptoms [17].

Mosaic trisomy 8 patients were also ascertained incidentally as a result of an infertility assessment [18]. One study reported that a 26-year-old woman with trisomy 8 mosaicism displayed mild mental retardation. She had a history of 5 pregnancies, four of which had poor outcomes; at the end of the last pregnancy, she gave birth to a girl at 39 weeks of gestation with normal karyotype [10]. The mechanism of chromosomal chimerism is mainly as follows: 1) mitotic postzygotic non-disjunction; 2) trisomy rescue; or 3) a new somatic mutation. If the mechanism of T8(M) is trisomy rescue or a new somatic mutation, the patients has normal reproductive abilities. However, if the mechanism of T8(M) is mitotic postzygotic non-disjunction, the patients may have normal reproductive abilities. Thus, in the process of meiosis, although individuals might generate different karyotypes of gametes, only the gamete with 23, X can undergo normal meiosis and survive, and 23,X does not exhibit an increasing trend in significant chromosomal abnormalities in the offspring of patients [19]. Therefore, further prenatal diagnosis is especially important for such patients.

Besides, the CMA analysis of this patient showed a loss of heterozygosity(LOH) in 11p15.5. A paternal uniparental disomy (UPD) in 11p15.5 region often cause a Beckwith-Wiedemann syndrome (BWS, MIM#130650). BWS is characterized by hemihypertrophy, macrosomia, macroglossia, organomegaly, hyperinsulinism, omphalocele/umbilical hernia, and distinct facial features [18]. A maternal UPD in this region may cause a Silver-Russell syndrome (SRS, MIM#180860). SRS is a congenital disorder typically associated with intrauterine and postnatal growth retardation [20]. In this study, this patient with the UPD in 11p15.5 region has no symptoms, which means it is a blood restricted UPD. Thus, in most cases diagnosis should be made by a combination of clinical features and molecular genetic findings.

In conclusion, in the present study, NIPT result suspected the pregnant woman was trisomy 8 mosaicism, and we further examined tissues from throat, cheeks and saliva, and cervical exfoliated cells, as well as blood, for NGS analysis. The results showed that the proportions of trisomy 8 in the different tissue samples were different; the saliva secreted by the salivary gland, which developed from ectoderm cells, showed partial trisomy 8, while the cervical cells, which developed from the mesoderm and blood, showed complete trisomy 8. The karyotype results showed that the fetal karyotype was 46,XX. Color ultrasonography showed that there was no abnormality and that the development of the fetus was normal, and a normal healthy female baby was born at term with the chromosome 46,XX.

## Data Availability

The datasets used or analysed during the current study are available from the corresponding author on reasonable request.

## Abbreviations

CML: Chronic myelogenous leukemia
MDS: Myelodysplastic syndrome
NGS: Next generation sequencing
NIPT: Noninvasive prenatal testing
NT: Nuchal translucency
T8MS: Trisomy 8 mosaicism syndrome
